# Case report: Hepatic inflammatory pseudotumor-like follicular dendritic cell sarcoma: a rare case and review of the literature

**DOI:** 10.3389/fmed.2023.1192998

**Published:** 2023-07-05

**Authors:** Shuai Yan, Zhiqiang Yue, Peng Zhang, Liuxia Yuan, Huixuan Wang, Fei Yin, Linglin Ju, Lin Chen, Weihua Cai, Yi Ni, Jinzhu Wu

**Affiliations:** ^1^School of Medicine, Nantong University, Nantong, Jiangsu, China; ^2^Institute of Liver Diseases, Affiliated Nantong Hospital 3 of Nantong University, Nantong, Jiangsu, China; ^3^Department of Nail and Breast Surgery, Affiliated Nantong Hospital 3 of Nantong University, Nantong, Jiangsu, China; ^4^Department of Hepatobiliary Surgery, Affiliated Nantong Hospital 3 of Nantong University, Nantong, Jiangsu, China

**Keywords:** inflammatory pseudotumor, follicular dendritic cell, sarcoma, case report, hepatic

## Abstract

Inflammatory pseudotumor-like follicular dendritic cell sarcoma (IPT-like FDCS) is a rare subtype of follicular dendritic cell sarcoma (FDCS) that primarily occurs in the liver and spleen. The etiology of IPT-like FDCS is unknown, and it has nonspecific clinical manifestations, imaging performance and laboratory test results. Recently, a patient with IPT-like FDCS was admitted to our hospital because of abdominal distension and anemia. Over the past 3 years, the patient has been followed up after a liver mass was found in a physical examination. The lesion gradually enlarged and caused compression symptoms. In November 2022, a tumor with a diameter of approximately 20 cm was found in the right posterior lobe of the liver after abdominal enhanced Magnetic resonance imaging (MRI) in our hospital. Liver tumor biopsy before the operation revealed a large number of hyperplastic plasma cells and a small number of spindle cells, and the spindle cells were atypical. After a complete examination, the patient underwent liver resection. Pathology after surgery confirmed liver IPT-like FDCS.

## 1. Introduction

FDCS is an indolent malignancy first described in 1986 (1). IPT-like FDCS is a rare subtype of FDCS. Although the pathogenesis and etiology of IPT-like FDCS are currently unknown, Epstein-Barr virus (EBV) infection has been considered an important factor in its occurrence because almost all patients are accompanied by Epstein–Barr virus-encoded RNA (EBER+). Women are prone to this tumor, and the liver and spleen are the most common sites of occurrence. The diagnosis of IPT-like FDCS lacks specific imaging manifestations, clinical symptoms, and laboratory test results. It is difficult to diagnose this tumor before surgery, so the diagnosis of IPT-like FDCS mainly depends on pathology. At the same time, mutual identification with IPT, hepatocellular carcinoma (HCC) and FDCS is needed.

## 2. Literature review

We systematically searched the PubMed, EMBASE, and MEDLINE databases using the search terms “liver” and “inflammatory pseudoneomatoid” in combination with “follicular dendritic cell sarcoma” or “follicular dendritic cell tumor” in studies published between 1996 and 2023. We collated demographic, clinicopathological, and follow-up information (Tables 1, 2).

**Table 1:** Clinicopathologic features of the reported cases of hepatic inflammatory pseudotumor-like follicular dendritic cell sarcoma.

**References**	**Case**	**Age(y)/ gender**	**Presentation**	**Tumor size**	**Treatment**	**Outcome**	**Recurrence or metastasis**
Selves et al. (2)	1	68/F	Malaise, weight loss, anemia	11	Chemotherapy, right hepatectomy	Alive and well at 30 months	N
Shek et al. (3)	2	35/F	epigastric discomfort, fever, weight loss	20	Right hepatectomy	Recurrence for three times since 30th month, died at 95 months	Y
Cheuk et al. (4)	3	19/F	Right upper quadrant pain, weight loss, palpable abdominal mass	12	Partial hepatectomy	Alive and well at 40 months	N
	4	56/F	Gastrointesinal upset	15	Resection of right lobe of liver	Recurrence at 15, 27, 35 months respectively	Y
	5	40/F	Epigastric pain, weight loss	12.5	Left hepatectomy	Recurrence at 108 months	N
	6	49/F	Asymptomatic	4.2	Partial hepatectomy	Alive and well at 9 months	N
	7	37/M	Malaise, weight loss	15	Right trisegmentectomy	Alive and well at 42 months	N
	8	35/F	Epigastric discomfort, low-grade fever, and weight loss	20	Right hemihepatectomy	first recurrence in liver and right hemi diaphragm at 30 mo; second recurrence in liver at 40 mo; third recurrence in peritoneum and ascending colon at 60 mo; died at 95 mo from disseminated tumor in liver and peritoneum	Y
	9	31/F	Abdominal distension, weight loss	15	Right hemihepatectomy	Alive and well at 60 months	N
Chen et al. (5)	10	57/F	Epigastralgia, anemia	9.5	No surgery	Alive and well at 36 months	N
	11	51/F	Abdominal distension	12	Left lobectomy	Alive and well at 12 months	N
Bai et al. (6)	12	30/F	Asymptomatic	5.6	Right hepatectomy	Alive and well at 24 months	N
Granados et al. (7)	13	57/F	Abdominal pain, vomiting, dizziness	13	Partial hepatectomy	Alive and well at 24 months	N
Liu et al. (8)	14	59/F	Asymptomatic	6	Partial hepatectomy	Alive and well at 17 months	N
Martins et al. (9)	15	53/F	Abdominal pain, jaundice, anemia, fever	11.5	Left hepatectomy	Alive and well at 6months	N
Li et al. (10)	16	39/F	Asymptomatic	9	Hepatic lobectomy, Chemotherapy and limited resection after recurrence	Alive and well at 84 months, Recurrence at 12 months	Y
	17	50/M	Abdominal fullness, fatigue	3	Left hepatectomy and splenectomy	Alive and well at 17 months	N
	18	42/F	Abdominal pain	2	Partial hepatectomy	Alive and well at 36 months	N
	19	38/F	Fatigue, anorexia	8.5	Left hepatectomy	Alive and well at 11 months	N
You et al. (11)	20	43/M	Right upper quadrant pain, weight loss	20	Unresectable	NA	NA
Nguyen et al. (12)	21	57/F	Weight loss	NA	Observation	NA	NA
Levi et al. (13)	22	19/M	Paraneoplastic arthritis	6	Partial hepatectomy	NA	NA
Chen et al. (14)	23	28/F	Abdominal pain	6	Left lobectomy of liver	Recurrence at 48 months	Y
	24	48/M	Abdominal pain	23.3	Extended right hemihepatectomy	Alive and well at 23 months	N
	25	60/M	Asymptomatic	3	Wedge resection	Alive and well at 3 months	N
Zhang et al. (15)	26	19/F	Abdominal discomfort	6	Hepatic VII segmental resection	Alive and well at 12 months	N
Li et al. (16)	27	31/F	Asymptomatic	3.6	Hepatectomy	Alive and well at 26 months	N
	28	48/M	Asymptomatic	10	Hepatectomy	Alive and well at 10 months	N
Zhang et al. (17)	29	31/F	Anorexia	3.5	Laparoscopic right hepatectomy	Alive and well at 10 months	N
	30	48/M	Asymptomatic	10	Right hepatectomy	Alive and well at 2 months	N
Deng et al. (18)	31	67/F	Cough, expectoration	4	Hepatectomy	NA	NA
Ang et al. (19)	32	63/F	Fever, lethargy	13.4	Right hemihepatectomy	Alive and well at 48 months	N
Jin et al. (20)	33	38/M	Asymptomatic	12.4	Hepatectomy	NA	NA
Wu et al. (21)	34	45/M	Epigastric pain	6.7	Right hepatectomy	Alive and well at 9 months	N
Liu et al. (22)	35	61/M	Asymptomatic	4.2	Laparoscopic hepatectomy	Alive and well at 13 months	N
Xu et al. (23)	36	81/M	Asymptomatic	NA	Resection	Alive and well at 12 months	N
	37	53/M	Abdominal distension	NA	Resection	Alive and well at 24 months	N
Pascariu et al. (24)	38	34/F	Epigastric pain	6	Laparoscopic hepatectomy	2 months after reoperation	Y
Lu et al. (25)	39	55/F	Epigastric pain	14.5	Hepatectomy	Paravertebral metastasis and recurrence,60 months with PR	Y
Li et al. (26)	40	47/M	Right upper quadrant abdominal pain	20	Hepatectomy	Alive and well at 50 months	N
Ding et al. (27)	41	66/F	Abdominal pain	7	Hepatic segment VI and VII resection	Alive and well at 84 months	N
Fu et al. (28)	42	23/F	Asymptomatic	3	Laparoscopic left liver resection	Alive and well at 6 months	N
Zhang et al. (29)	43	35/F	Epigastric pain	1.5	Laparoscopic resection	NA	NA
Present case	44	55/F	Abdominal pain	20	Right Hepatectomy	Alive and well at 6 months	N

F, female; M, male; Y, yes; N, no; NA, not available.

**Table 2:** Results of immunohistochemical.

**Case**	**EBER**	**CD21**	**CD23**	**CD35**	**Plasma cell infiltration**
1	+	+	+	NA	Y
2	+	+	NA	+	N
3	+	+	-	+	Y
4	+	+	-	+	Y
5	+	+	-	+	Y
6	+	+	+	+	Y
7	+	+	+	+	Y
8	+	+	+	+	Y
9	+	+	-	+	Y
10	+	+	+	-	Y
11	+	+	+	-	N
12	+	+	NA	+	Y
13	+	+	+	NA	Y
14	+	+	+	+	Y
15	-	+	-	+	N
16	+	+	NA	+	Y
17	+	+	NA	+	Y
18	+	+	NA	+	Y
19	+	+	NA	+	Y
20	+	-	-	-	Y
21	+	NA	NA	NA	NA
22	+	NA	NA	NA	NA
23	+	+	+	+	Y
24	+	+	+	+	Y
25	+	+	+	+	Y
26	+	+	NA	+	N
27	+	+	+	NA	Y
28	+	+	NA	NA	Y
29	+	+	NA	NA	Y
30	+	+	NA	NA	Y
31	+	+	+	NA	Y
32	+	+	+	NA	Y
33	+	+	+	+	Y
34	+	+	NA	NA	Y
35	-	-	+	+	Y
36	NA	+	NA	+	NA
37	NA	+	NA	+	NA
38	+	+	NA	+	Y
39	+	+	+	-	Y
40	+	+	-	+	Y
41	+	+	NA	+	Y
42	+	+	NA	+	N
43	+	+	+	NA	Y
44	+	+	+	+	Y
Positive rate	90.9% (40/44)	90.9% (40/44)	43.1% (19/44)	63.6% (28/44)	79.5% (35/44)

Y, yes; N, no; NA, not available.

## 3. Case presentation

**Figure 1:**
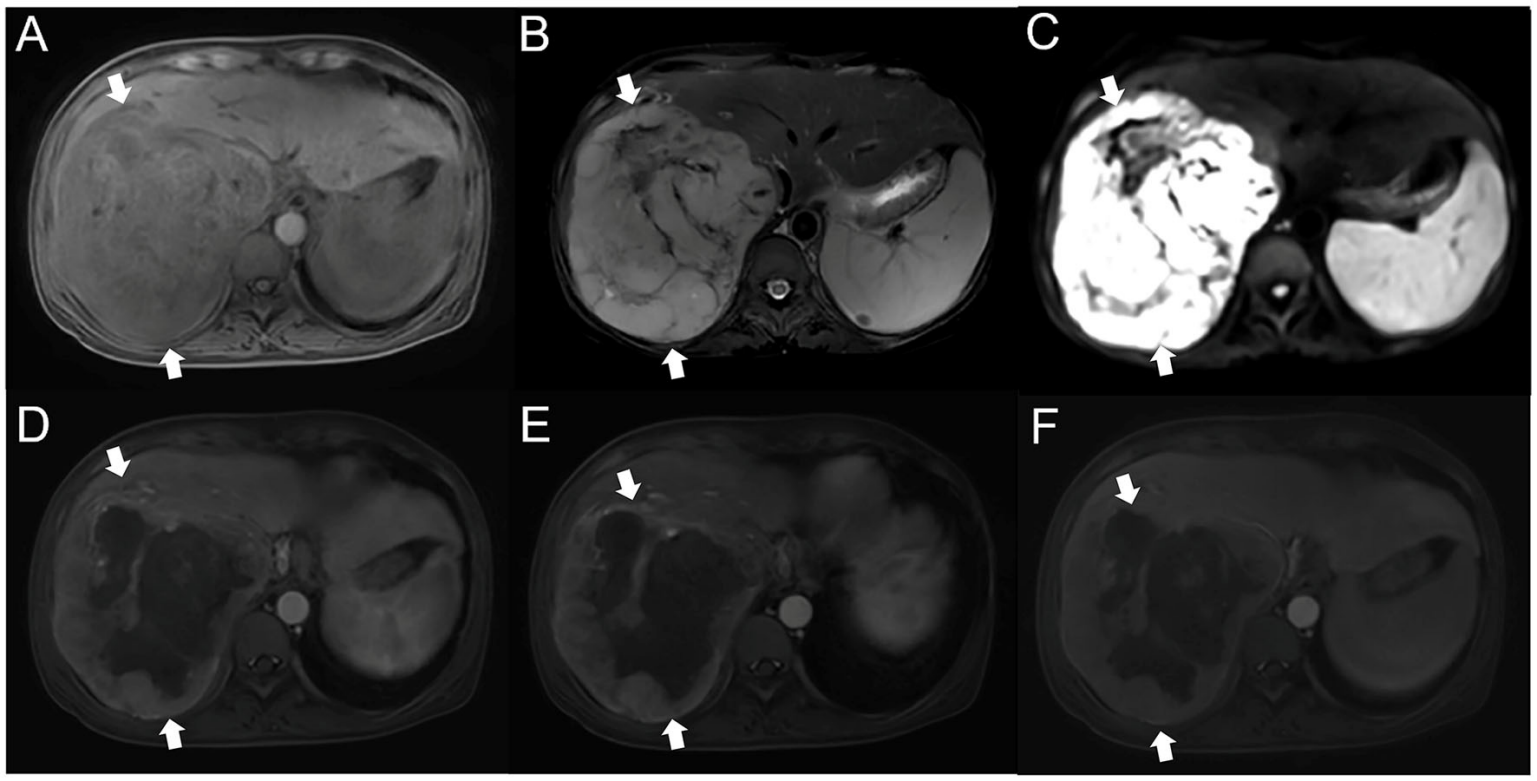
MRI of the patient's liver **(A)**. T1WI showed the presence of clumpy and other low-confounding signal shadows in the right lobe of the liver **(B)**. Most of the lesion site on T2WI had slightly higher signal shadows, with irregular bars of low signal shadows inside **(C)**. On DWI, the lesion site was unevenly hyperintense **(D)**. Contrast-enhanced MRI showed that the edge of the arterial lesion was strengthened, with significant punctate strengthening, and large areas of low intensity were seen in the lesion **(E, F)**. The lesion site in the portal phase and delayed phase had a clear marginal contour and mild internal delayed intensification. MRI, magnetic resonance imaging; T1WI, T1 weighted image; T2WI, T2 weighted image; DWI, diffusion weighted imaging.

We report the case of a 55-year-old female patient. She is a farmer and has no history of exposure to industrial poisons, dust, or radioactive substances. Physical examination 3 years prior found a liver mass. Liver tumor biopsy performed in another hospital revealed an extramedullary plasmacytoma. She was treated with methotrexate, prednisone, hydroxychloroquine, iguratimod, and calcitriol, but the treatment was ineffective. The patient came to our hospital for treatment due to symptoms of abdominal distension and anemia. Physical examination revealed the following: high distension in the right upper quadrant; a palpable mass that was inactive, hard, and poorly circumscribed; and tenderness under the xiphoid process and subcostal margin of the right upper quadrant. Conventional and contrast-enhanced MRI showed a 20 cm diameter mass in the right posterior lobe of the liver (Figure 1). T1WI showed that most of the lesion site had iso-low intensity signal shadows, while T2WI showed that most of the lesion site had slightly higher signal shadows with low focal shadows. DWI showed that the lesion had a high signal as a whole, and irregular low signal stripes were seen inside. In the arterial phase, the edge of the lesion area was enhanced, and there was a patchy low signal area inside. During the portal phase, the signal of the lesion was slightly higher, and there was a patchy mildly enhanced area inside the lesion during the delayed scan.

**Figure 2:**
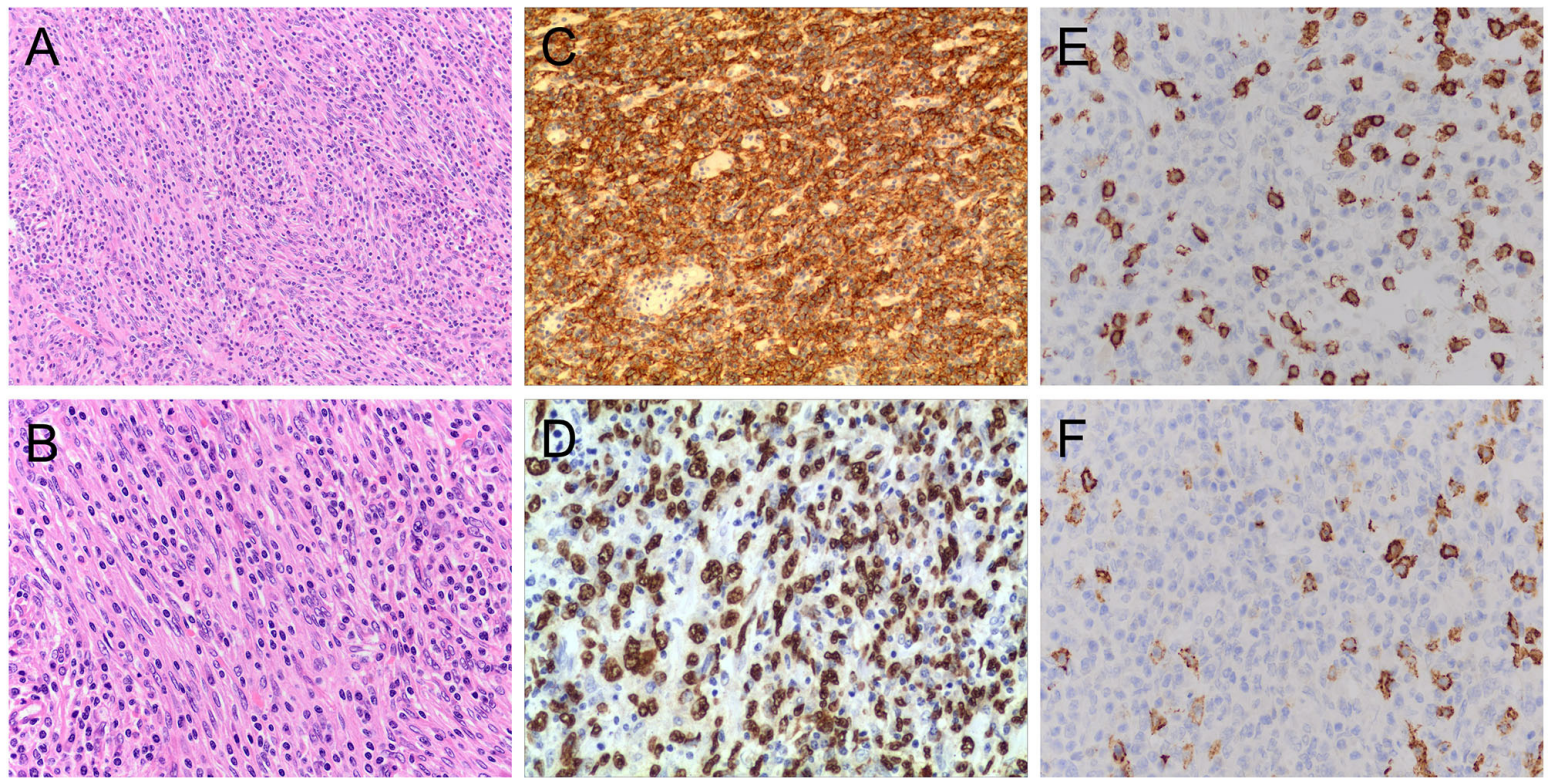
Pathological characteristics of the tumor. H&E stained image showing that the tumor contained a mixture of proliferative spindle cells and a large number of small lymphocytes and plasma cells. Hematoxylin and eosin stain, original magnification 200X **(A)** and 400X **(B)**. Immunohistochemistry showed that the neoplastic cells were strongly positive for CD23 **(C)** and EBER staining **(D)**. Magnification 200X **(C)** and 400X **(D)**. In CD3 **(E)** and CD20 **(F)** staining, the oval tumor cells were negative, and there were positive T cells and B cells infiltrated in the background.Magnification 400X **(E)** and 400X **(F)**. H&E, hematoxylin and eosin;EBER, Epstein–Barr virus-encoded RNA.

Her bone marrow biopsy was negative. Her liver mass biopsy showed a large number of hyperplastic plasma cells and a small number of atypical spindle cells. SFLVRS 78.2% (height 155 cm, weight 47 kg, remnant liver volume 791 ml). The patient underwent right hemihepatectomy. The operation is open, and we carefully explored the abdominal cavity during the operation and found no abnormal lesions of the stomach, intestines, gallbladder, pancreas, or spleen. The main trunk and left and right branches of the portal vein of the liver were not significantly embolized. The total operation time is 4 h and 35 min, and the blood loss during the operation is 1,000 ml. The overall recovery of the patient was good, and there were no complications such as bleeding and bile leakage. The patient was discharged after 13 days. Pathological analysis of surgical specimens showed that the tumor was composed of proliferating spindle cells arranged in an interlaced manner, with weakly acidophilic cytoplasm, oval nuclei, flocculent or fine-grained chromatin, small and clear nucleoli, and increased plasma in the background (Figure 2). The immunohistochemical results were CD21(+), CD23(+), CD35(+), CD138(–), CD20(–), CD3(–), CD30(–), CD79α(–), Ki67(~20% +), Kappa(+), Lambda(+), MUM-1(–), ALK(–), CD117(-), Desmin(–), S-100(–), AE1/AE3(-) and D2-40 (partially +). In situ hybridization showed EBER positivity (Figure 2). The final pathological diagnosis was IPT-like FDCS. No tumor recurrence was found during the 3-month follow-up.

## 4. Discussion

### 4.1. FDCS

FDCS is a rare sarcomatoid hyperplasia of follicular dendritic cells first described by Monda et al. (1). In the 4th edition of the World Health Organization (WHO) Classification of Tumors of Hematopoietic and Lymphoid Tissues revised in 2017 (30), based on common functional properties, FDCS and tumors derived from histiocytes and hematopoietic dendritic cells are classified as one class, such as antigen presenting cells (APCs). The tumor behavior of FDCS is similar to that of moderate-grade sarcomas, with a greater risk of recurrence and metastasis. Some studies suggest that FDCS has the following clinicopathological features: 1. young age ( ≤ 40 years); 2. no plasma cell infiltration; 3. large tumor size (≥6 cm); 4. high mitotic count (≥5/10 high power field); and 5. cytologically atypical features associated with poor prognosis of tumors (30, 31). We counted that in the previously reported cases of IPT-like FDCS of the liver, 15.9% (7/44) of the patients had metastases, of which 100% (7/7) had a tumor diameter ≥6 cm, and 14.2% (1/7) of the patients had no plasma cell infiltration.

### 4.2. IPT-like FDCS

In addition to classical FDCS, there is a form of extranodal FDCS associated with EBV infection. Because this form is more histologically similar to inflammatory pseudotumor than to sarcoma in classical FDCS, it is called IPT-like FDCS (4). With morphological and clinical features in between those of inflammatory pseudotumor and follicular dendritic cell neoplasm, IPT-like FDCS was identified as a unique variant in 2001 and was included in the FDCS diagnostic category (4, 32). In terms of tumor behavior, IPT-like FDCS is more inclined to behave as a low-grade malignant tumor with a good prognosis, which is more indolent and has a lower chance of recurrence and metastasis (33). In our literature review, 15.9% (7/44) of patients with liver IPT-like FDCS had tumor recurrence or metastasis. In IPT-like FDCS, which occurs in the liver, the median age of patients is 48 years (19–81), and it is significantly dominant in women (1:2.14). Patients are generally asymptomatic or have abdominal distension and abdominal pain (65.9%, 29/44), and a small number of patients have accompanying symptoms, such as weight loss, anemia, and fever. It is also worth mentioning that one patient with paraneoplastic arthritis was identified in our literature review. This is consistent with previous reports suggesting that IPT-like FDCS can exhibit paraneoplastic arthritis in rare cases (13).

By comparing IPT-like FDCS with classical FDCS, it can be found that EBV infection occurs relatively rarely in patients with classical FDCS, while it is closely related in the IPT-like variant of FDCS (34). In our literature review, 40 patients had EBER(+) liver IPT-like FDCS, which accounted for 90.9% (40/44). From the perspective of mechanism, first, EBV infection may lead to pathological changes such as vascular proliferation, damage and inflammation, which is especially obvious in the monoclonal hyperplasia environment of tumors (12). Second, the receptor CD21 of EBV is highly expressed in FDCs, which leads to EBV entering cells more conveniently (35, 36). Furthermore, the presence of monoclonal EBV genomes in spindle cells suggests that EBV infection is a transformation event of FDCs, and some studies have even suggested that the occurrence of the entire IPT-like FDCS may originate from a cell infected with EBV, such as Burkitt lymphoma and nasopharyngeal carcinoma (3). Finally, in hepatic IPT-like FDCS, tumor cells exhibit multiple immunophenotypic features, suggesting that they may be derived from a variety of common mesenchymal stem cells that differentiate into FDCS by acquiring CD21 and CD35 (37), suggesting that EBV infection may induce tumor transformation of mesenchymal cell-derived FDCs and express CD21 and CD35 (34, 37). Although the specific pathogenesis of EBV in IPT-like FDCS remains unknown, EBV infection can now be considered the main means of differentiating the two FDCSs in light of the above studies.

### 4.3. Diagnosis

Currently, the diagnosis of IPT-like FDCS is often difficult; not only does it require ancillary tests, including imaging, cytological features, immunohistochemical testing of FDC markers, and EBER, but the scarcity of cases and lack of specific clinical symptoms add to the challenge of diagnosing IPT-like FDCS (38). We found that the MRI feature of IPT-like FDCS is a well-circumscribed soft-tissue mass with a fibrous capsule-like structure. MRI shows progressive enhancement of the parenchyma, suggesting rich blood supply in the tumor parenchyma and clearer boundaries in the arterial phase. On DWI, the solid part of the tumor is restricted in spread, while the area of liquefaction necrosis is not restricted, indicating that the solid part of the tumor has a higher density of tumor cells (39). In this case, the signal in the solid part of the tumor was inhomogeneous on MRI, and inhomogeneous hyperintensity was seen on DWI. Since MRI signal intensity varies with the composition of the parenchymal fraction (40), it is considered that this may be related to microhemorrhages in the parenchyma or the uneven distribution of inflammatory cells.

The diagnosis of IPT-like FDCS also requires cytological and histological characteristics (7). In these two respects, classical FDCS is highly similar to IPT-like FDCS. Tumor cells can appear atypical, usually fusiform, ovoid, or polygonal, and form star-shaped, fascicular, or trabecular arrangements (22). The cells also have eosinophilic cytoplasm, oval or irregular nuclei, darker chromatin, and prominent nucleoli (7, 41). However, IPT-like FDCS has a very prominent inflammatory component, mainly including lymphocytes (B and T cells), eosinophils, plasma cells, and rare epithelioid histiocytes. Tumor cells are usually masked by inflammatory infiltrates (4, 10). Because of this, IPT-like FDCS is often mistakenly identified as an inflammatory response or IPT or a variety of other tumors (42).

In addition to observing cell morphology and tissue characteristics, diagnosing IPT-like FDCS typically requires a variety of FDC markers, including CD21, CD23, CD35, CXCL-13, D2-40, clusterin, fascin, epidermal growth factor receptor, and CNA42 (43, 44). In the data we collected, the positive rate of CD21 was 90.9% (40/44), CD23 was 43.1% (19/44), and CD35 was 63.6% (28/44). This shows that among many markers, CD21 and CD35 have the highest specificity (45). Positive staining for Smooth muscle actin (SMA), vimentin, S100, and CD68 was nonspecific (33, 46). In addition, other sensitive and specific markers, such as γ-synuclein and desmoplakin, may serve as auxiliary markers (31, 47). Recent studies have shown that FDCS has a significantly higher positive rate in immunohistochemical analysis of SSTR2a than CD21 and CD35 in conventional subtypes, and it is negative in all IPT-like variants (48). Therefore, SSTR2 is expected to be a highly sensitive diagnostic marker to distinguish FDCS from IPT-like FDCS (27).

### 4.4. Differential diagnosis

In the differential diagnosis of IPT-like FDCS, first, hepatic IPT-like FDCS may be misdiagnosed as IPT at the initial evaluation. In terms of clinical symptoms, both diseases may show a severe inflammatory response. Their differential diagnosis can be based on tumor behavior, FDC markers, EBER and ALK expression (22).

Second, hepatic IPT-like FDCS should also be differentiated from HCC with internal necrosis by imaging findings, clinical features, and laboratory tests. HCC often has peripheral invasion with cirrhosis and portal hypertension and may be accompanied by carcinoma thrombosis. HCC is the most common liver malignancy. Chronic viral hepatitis and elevated AFP often indicate HCC (22). If IPT-like FDCS is suspected, in addition to the identification of clinical manifestations and laboratory tests, it is important to perform routine biopsy. Mass aspiration biopsy is a feasible preoperative diagnostic method for IPT-like FDCS, but due to the small number of tissues obtained by puncture, there are many false-negative cases, and because of the influence of plasma cell infiltration, there will be a possibility of misdiagnosis of extramedullary plasmacytoma. Therefore, its definitive diagnosis mainly depends on immunohistochemistry and EBER of the tumor specimen obtained by surgery.

Finally, in the differential diagnosis of FDCS, in addition to being positive for EBV in EBER, the noninvasive biological behavior of IPT-like FDCS can further support the relatively inert and slow-growing characteristics of IPT-like FDCS (49), and it has an auxiliary effect on the diagnosis of IPT-like FDCS. Clinicians can better distinguish FDCS from IPT-like FDCS by performing a pathological examination of surgically removed tissue samples (28). Moreover, IPT-like FDCS shows chronic inflammation characteristics different from FDCS in imaging.

### 4.5. Treatment

Due to the low incidence and variable clinical course of IPT-like FDCS, the best treatment options are currently uncertain. The usual clinical treatment is surgical resection, and the value of postoperative chemotherapy and radiotherapy is uncertain (38). Among the data we collected, only 4.5% (2/44) of the patients received chemotherapy or radiotherapy, and no recurrence, metastasis or death was found in the follow-up of these 2 patients. It has been suggested that chemotherapy or radiotherapy is needed for advanced and incompletely resected tumors, which can help achieve relatively effective tumor control. Shinagare et al. (50) described a case of FDCS with a liver size of 11 cm, but the remnant liver volume was not enough to meet the surgical resection criteria. After 4 cycles of cyclophosphamide, doxorubicin, vincristine and prednisone (CHOP) chemotherapy and portal vein embolization, the tumor was successfully resected. Notably, PD1/PD-L1 checkpoints have been reported to play an important role in the regulation of the immune environment of FDCS, and elevated levels of PD-1 and PD-L1 expression have been found in FDCS (51–53). Given that immunotherapy has shown striking therapeutic effects in recent years (54, 55), PD-1/PD-L1 immune checkpoint inhibitors may be a promising neoadjuvant therapy for unresectable FDCS.

### 4.6. Limitations

Our review has some limitations. First, the follow-up of patients was relatively short, making it difficult to assess long-term prognosis. Second, since the diagnosis of IPT-like FDCS was derived from postoperative pathology, some preoperative laboratory tests for EBV-related diseases were not performed, which could not further strengthen the results of EBER. Finally, our collection of data was limited by a small sample size and does not characterize the disease. Therefore, more in-depth and comprehensive studies are needed to determine the pathogenic mechanism of hepatic IPT-like FDCS, its association with EBV, and its treatment options.

## 5. Conclusion

IPT-like FDCS is a rare tumor, but it has been reported with increasing frequency. Clinicians must keep the differential diagnosis in mind and consider additional disease possibilities when encountering liver tumors showing unusual histologic features. At present, surgical resection is the best way to treat IPT-like FDCS, but there remains a lack of strong evidence for the effect of chemotherapy. At the same time, the pathogenesis and etiology of IPT-like FDCS need further study.

## Data availability statement

The original contributions presented in the study are included in the article/supplementary material, further inquiries can be directed to the corresponding authors.

## Ethics statement

The studies involving human participants were reviewed and approved by Institutional Review Board review (EK2022023) at the Affiliated Nantong Hospital 3 of Nantong University in Nantong, China. The patients/participants provided their written informed consent to participate in this study. Written informed consent was obtained from the individual(s) for the publication of any potentially identifiable images or data included in this article.

## Author contributions

JW and WC were the patient's physician and responsible for the revision of the manuscript for important intellectual content. SY, ZY, LY, and HW reviewed the literature and contributed to drafting the manuscript. YN and FY performed the radiographic analysis. PZ, LJ, and LC conceptualized and designed the study, coordinated and supervised data collection, and critically reviewed the manuscript for important intellectual content. All authors issued final approval for the version to be submitted for publication.
